# Environmental DNA Reveals the Impact of Submarine Groundwater Discharge on the Spatial Variability of Coastal Fish Diversity

**DOI:** 10.3390/biology13080609

**Published:** 2024-08-11

**Authors:** Nguyen Hong Nhat, Mitsuyo Saito, Shin-ichi Onodera, Mayuko Hamada, Fujio Hyodo, Hideaki Nagare

**Affiliations:** 1 Graduate School of Environmental and Life Science, Okayama University, Okayama 7008530, Japan; p1qd3i9i@s.okayama-u.ac.jp; 2 Faculty of Technology—Engineering—Environment, An Giang University, Vietnam National University—Ho Chi Minh City, Ho Chi Minh City 880000, Vietnam; 3 Graduate School of Advanced Science and Engineering, Hiroshima University, Higashi Hiroshima 7398521, Japan; sonodera@hiroshima-u.ac.jp; 4 Ushimado Marine Institute (UMI), Graduate School of Environment, Life, Natural Science and Technology, Okayama University, Okayama 7014303, Japan; hamadam@okayama-u.ac.jp; 5 Faculty of Environmental, Life, Natural Science and Technology, Okayama University, Okayama 7008530, Japan; fhyodo@cc.okayama-u.ac.jp (F.H.); nagare-h@okayama-u.ac.jp (H.N.)

**Keywords:** eDNA, metabarcoding, biodiversity, functional diversity, taxonomic diversity, spatial patterns, environmental variables

## Abstract

**Simple Summary:**

In environments characterized by small-scale islands, terrestrial inputs, like freshwater discharges, can significantly impact fish communities compared to ocean currents. While the influence of river discharge on marine biodiversity is well documented, the effects of submarine groundwater discharge on nearshore fish communities are less understood. Here, our results provide evidence that submarine groundwater discharge crucially influences coastal fish diversity by altering salinity and nutrient supply. This study also showed that fish community composition varies considerably over a small spatial scale, reflecting habitat partitioning on the target island.

**Abstract:**

Submarine groundwater discharge (SGD) has recently been recognized as an influential factor in coastal ecosystems; however, little research has been conducted on its effects on coastal fish diversity. To investigate the relationship between SGD and fish diversity, we conducted a survey at the coastal island scale using the environmental DNA (eDNA) method. Our findings indicate that fish species richness and functional richness peak at stations with high SGD. Environmental variables, such as salinity, dissolved inorganic nitrogen (DIN) concentration, and SGD, significantly influence fish diversity. Carnivore fish richness was negatively correlated with salinity, while planktivore fish richness was positively correlated. Additionally, SGD and DIN concentrations were found to be crucial in shaping omnivorous and pelagic communities, respectively. This study highlights the role of SGD in enhancing nutrient conditions favorable for diverse fish communities and demonstrates the effectiveness of eDNA metabarcoding for rapid marine biodiversity assessment. These findings provide valuable insights for coastal ecosystem monitoring and management.

## 1. Introduction

Surface and subsurface water discharge to coastal areas is essential for supporting biodiversity by supplying the necessary nutrients for the growth and reproduction of marine organisms [1] and creating an optimal habitat environment, such as for temperature and salinity [2,3]. Several marine ecological studies have shown how river discharge (RD) affects the biological community structure and supports production in coastal areas [4]. However, few studies have focused on the impacts of submarine groundwater discharge (SGD) on coastal ecosystems [5]. Some studies have revealed that salinity changes caused by SGD influence the macro-benthic community composition [3,6]. Salinity has also been identified as a key variable for fish species distribution; for example, high salinity negatively affects the metabolism and feeding behavior of some fish species [7]. Moreover, SGD usually contains a higher concentration of nutrients than surface water, which can significantly affect phytoplankton growth and species changes in phytoplankton [4,5,6,7,8]. Fujita et al. [8] confirmed a positive relationship between SGD-derived nutrient loading and elevated benthic primary production, affecting the growth of juvenile marbled soles (*Pseudopleuronectes yokohamae*). Lilkendey et al. [9] demonstrated the physiological effects of fresh SGD on the augmented growth of juvenile gray demoiselle (*Chrysiptera glauca*) by providing optimal salinity and low pH conditions. These studies suggest that SGD is important for specific fish species by providing optimal habitats and nutrients to increase primary and secondary production. However, the effects of SGD on coastal fish diversity are poorly understood. A spatial evaluation of the relationship between SGD and fish communities would provide useful information to examine this.

Environmental DNA (eDNA) has emerged as a promising tool for investigating marine biodiversity via rapid and integrative biomonitoring [10]. eDNA is generated when organisms release genetic material into their environment and can be isolated, extracted, and sequenced using metabarcoding techniques [11,12,13,14]. This approach has shown superiority in species detection, particularly for cryptic and rare species, while being non-invasive and cost-effective [15,16,17], offering advantages over traditional surveys [10,18,19]. Recent advancements in eDNA technology and methodology have proven highly accurate in predicting the spatial distribution of species [20]. eDNA can be used to distinguish the taxonomic diversity of various habitats and quickly assess changes in biodiversity at the ecosystem level [17]. However, relying solely on individual taxonomic diversity is insufficient to reveal community variations [21]. Integrating taxonomic and functional diversity offers deeper insight into the intricate relationships between species and their environments [22,23,24]. Functional diversity, including feeding habits, swimming abilities, and habitat preferences, provides a comprehensive understanding of the responses of fish communities to various environmental conditions [2,25,26,27]. Hence, using a reliable integrated eDNA-functional biomonitoring method is valuable for fish biodiversity assessment and comprehensive impact evaluation of SGD.

Therefore, in this study, we examined the impact of SGD on the spatial patterns of fish diversity on a coastal island in western Japan where the spatial variability of SGD was confirmed [28]. To investigate the relationship between SGD and the taxonomic and functional diversity of fish, we used eDNA at an island scale.

## 2. Materials and Methods

### 2.1. Study Area Information

**Figure 1:**
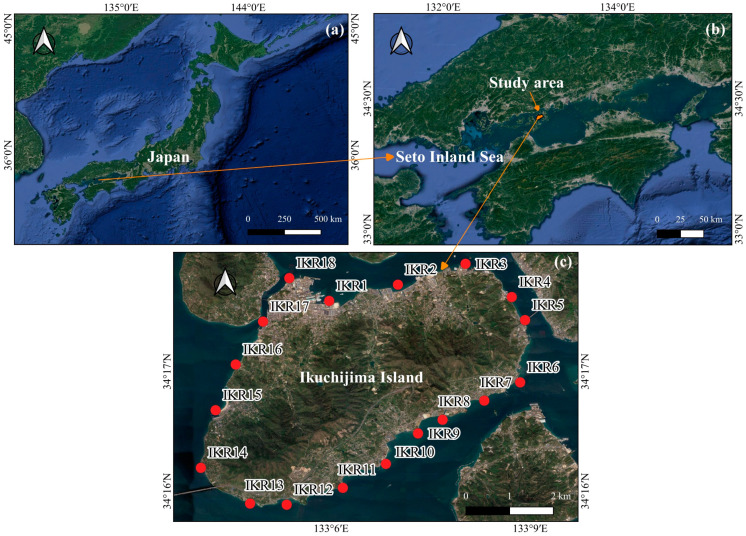
Map showing the study area, with red dots indicating sampling stations. (**a**) Japan; (**b**) Seto Inland Sea and (**c**) Ikuchijima Island.

Ikuchijima Island (34°16′ N, 133°6′ E) is centrally located within the Seto Inland Sea (SIS) of western Japan. This island covers an area of 31.2 km^2^, with a perimeter of 33.6 km and a maximum elevation of 472 m. The topographic gradient is steeper in the western, southern, and eastern parts of the island than in the northern areas [28]. Annual average temperature and precipitation are 16 °C and 1100 mm [28,29]. The coastal zone surrounding the island is characterized by a high level of fish diversity of numerous species [29,30,31]. In November 2022, we surveyed 18 sampling stations (IKR1–18) in the seawater of the island. The numbering and distribution of each sampling station are shown in Figure 1c.

### 2.2. Sampling Collection

Seawater samples were collected from a boat by slowly circling around the target island. We used the geo-coordinates of each sampling station, directly measured at the study area with a GPS device for spatial variables. For collecting eDNA, two bottles of 1-L water sample per site (two liters) were collected from the surface using polypropylene bottles in duplicate for each station, immediately adding 1 mL of 10% benzalkonium chloride solution (Osvan 10%, Nihon Pharmaceutical Co., Ltd., Tokyo, Japan) to prevent eDNA degradation by bacteria, following the sampling manual of the eDNA society [32]. Blanks (negative controls) were set up at the study area by sterilized deionized water in the same manner to examine for contamination during the field survey. Field water samples and distilled water were kept on ice during sampling and transportation until filtration. Within 24 h after collection, each 1 L seawater sample was filtered through a Sterivex-HV filter with pore size 0.45 μm (Merck Millipore, Burlington, MA, USA) and was directly immersed in 1.5 mL of RNAlater (Thermo fisher Scientific, Waltham, MA, USA) to avoid degradation of DNA, then filtered samples were transported to the laboratory at −20 °C for further eDNA analysis.

In addition, a further 100 mL of seawater samples for each station were collected with three replicates and transported to the laboratory at 4 °C for nutrients analysis, including ammonium nitrogen (NH_4_-N, mg/L), nitrite (NO_2_-N, mg/L), nitrate (NO_3_-N, mg/L), phosphate (PO_4_-P, mg/L) and silicate (Si(OH)_4_-Si, mg/L). Nutrient analyses were performed using an auto-analyzer (swAAt, BLTEC). We define dissolved inorganic nitrogen (DIN) as the sum of ammonium nitrogen (NH_4_-N, mg/L), nitrite (NO_2_-N, mg/L), and nitrate (NO_3_-N, mg/L). Phosphate (PO_4_-P, mg/L) is reported as dissolved inorganic phosphorus (DIP), while silicate (Si(OH)_4_-Si, mg/L) is reported as dissolved silica (DSi). We also collected particulate organic matter (POM, an indicator of phytoplankton) of 0.7–125 µm from the surface seawater of each station [33]. Those samples were filtered on pre-combusted glass-fiber filters (Whatman GF/F type, 450 °C, 2 h) after sieving through a 125 µm mesh sieve [33]. While collecting seawater samples, a portable data logger (CTD-Diver, vanEssen Instruments, Delft, The Netherlands) was employed to record seawater temperatures and salinity at each station.

### 2.3. Environmental DNA Analysis, Taxonomic Verification

eDNA experiments, including extraction of eDNA and polymerase chain reaction (PCR) of technology, were conducted in a dedicated laboratory, PCR-free to avoid any contamination of eDNA samples. Total DNA was extracted by using a DNeasy Blood and Tissue Kit (Qiagen, Hilden, Germany) [34]. After purification, DNA was eluted with 100 μL elution buffer (buffer AE) and stored at −20 °C. The amplicon libraries of the spatial 12S rRNA region were constructed using the universal Mifish primer sets (MiFish-E-F/R-v2:MiFish-U-F/R:MiFish-U2-F/R = 1:2:1) [35]. KAPA HiFi HotStart ReadyMix (KAPA Biosystems, Wilmington, MA, USA) for amplification of eDNA, GeneRead Size Selection Kit (Qiagen, Hilden, Germany) and Agencourt AMPure XP (Beckman Coulter, Tokyo, Japan) for purification of first and second PCR products, respectively, were used according to the manufacturer’s protocol. In addition to the field/filtration blanks, PCR-negative controls were made using distilled water instead of eDNA during PCR. Purified PCR products were quantified using a Qubit 2.0 fluorometer and dsDNA HS Assay Kit (Thermo Fisher Scientific, Waltham, MA, USA), and TapeStation 4150 and DNA High Sensitivity D1000 (Agilent, Santa Clara, CA, USA). The obtained sequencing libraries were quantified using a Qubit 2.0 fluorometer and dsDNA HS Assay Kit (Thermo Fisher Scientific) and pooled in equal concentrations. Libraries were sequenced using an Illumina MiSeq system with 600-cycle chemistry (2 × 300 bp paired-end sequencing using the MiSeq Reagent Kit v3) (Illumina, San Diego, CA, USA).

Sequence pairs of all samples were performed following Miya et al. [35] and using the publicly available bioinformatics pipeline, Mifish Pipeline [36,37]. All sequencing data underwent the following processes: quality control, trimming, assembled cleaned, N-base and length filtering (229 ± 25 bp by default), primer removal, clustering and species-level assignment. The zero-radius operation taxonomic units table (ZOTUs) was obtained and queried against the Mitofish database (fish mitochondrial genome database) with threshold of identify 97% and e-value 10^−5^ [37,38]. After this step, a taxonomic database was made and classified based on information obtained from FishBase.

### 2.4. Functional Traits Verification

Functional traits of the identified fish species, such as feeding habits and depth levels, were compiled from existing literature databases and FishBase (Table 1).

### 2.5. Evaluation of Spatial Variability of SGD

Recently, DSi has become well known as a useful geochemical tracer for estimating SGD [41,42,43]. SGD typically contains high concentrations of DSi, making DSi a valuable addition to the conventional tracers, such as ^222^Rn or Ra [44,45]. Here, we applied DSi balance model to estimate the spatial variation in SGD on the island [42,46]. The entire island was divided into 18 boxes based on the locations of DSi sampling stations. The SGD for each box was then calculated by using the DSi balance equation, allowing us to assess the spatial distribution of SGD across the island. At steady state, sources of DSi in a system can be included, such as diffusion from bottom sediments, SGD, RD and mixing with offshore seawater. The mass balance equation for DSi can then be written as follows [41,42,46].

(1) $$F_{R}^{Si}+F_{Diff}^{Si}+C_{GW}^{Si}{\times A}_{Bott}\times \varphi_{SGD}+C_{EX}^{Si}\times V_{s}\times \delta_{Mix}=0$$

where $F_{R}^{Si}$ is the DSi flux by RD (mmol/d), which was estimated from river flow rate ($Q_{R}$) and DSi concentrations ($C_{R}^{Si}$) of river water ($F_{R}^{Si}=Q_{R}\;\times C_{R}^{Si})$; $F_{Diff}^{Si}=R_{G}^{Si}\times A_{Bott}$ is the flux due to diffusion from bottom sediments (mmol/d), $R_{G}^{Si}$ is the regeneration rate of DSi (mmol/m^2^d), $A_{Bott}$ is the bottom area of each sections (m^2^); $C_{GW}^{Si}\times A_{Bott}\times \varphi_{SGD}$ is the flux of DSi from SGD ($F_{SGD}^{Si}$), $C_{GW}^{Si}$ is the DSi concentration of groundwater end-member (mmol/m^3^), $\varphi_{SGD}$ is the seepage rate of groundwater (m/d); $C_{EX}^{Si}\times V_{s}\times \delta_{Mix}$ is the mixing with offshore seawater ($F_{Mix}^{Si}$), $C_{EX}^{Si}$ is the concentration of DSi in each section ($C_{SW}^{Si}$) subtracted for the offshore seawater concentration ($C_{O}^{Si}$), $V_{s}$ is the volume of each section (m^3^), $\delta_{Mix}$ is the rate at which seawater mixed with offshore seawater (d^−1^).

### 2.6. Data Analysis and Statistics

In this study, QGis software version 3.16.16 was used to visualize the spatial variation of SGD, alpha diversity, fish species composition and functional traits. The alpha diversity index, including species richness and functional richness, was calculated for characterizing fish diversity by R package vegan and mFD [47]. In a community, functional richness (FRic) indicates the number of ecological niches occupied by species. An increase in FRic indicates a more efficient use of ecological space, which reflects the stability of the community and resistance to invasion [48]. The fish reads were transformed to presence/absence data before undergoing statistical analysis. The Spearman correlation analysis performed using the linkET package (mantel test function) in R to determine the correlations between environmental variables [49], expresses statistical significance as a *p*-value less than 0.05. Environmental variables that were not normally distributed were transformed by using log transformations and were then standardized depending on the implementation of each analysis process [50]. To identify the major variables explaining the pattern in species richness, functional richness among the target area, we used the generalized linear models (GLMs) with a Poisson distribution and log-link function using the *glm* function [51]. *Ggpairs* function was used to determine the covariation among environmental variables, and the highly correlated variables (r > 0.5) were removed to minimize collinearity in the subsequent analyses. For each response variable, we first constructed a full model that comprised of all the environmental variables (i.e., SGD, RD, etc.). Then the *dredge* function in the MuMIn package was used for model selection [51]. The best models were selected using Akaike’s information criterion (AIC), and the best models have the lower value of AIC [51]. Distance-based redundancy analysis (dbRDA) was conducted to assess environmental factors affecting fish communities’ composition, with ordination biplot explaining the relationships between significant environmental variables and these patterns by R package vegan [52]. To reduce the presence of covariance among the environmental variables, variance inflation factors (VIF) were calculated to assess collinearity among predictors. VIF values were all <2, suggesting non-collinearities [50]. Then some variables with high correlation were removed and only the selected variables were input in the final model. Data underwent a three-step process: first, a community dissimilarity matrix is calculated using the Jaccard dissimilarity matrix, which derived from presence/absence species level data. Next, we performed dbRDA on dissimilarity matrix by using *capscale* function. Then, the permutation tests were separately performed on the axes of the model and the full model (number of permutations: 999). We analyzed the total fish species community and functional communities separately. All statistical analyses were carried out in R version 4.2.0.

## 3. Results

### 3.1. Spatial Variability of Fish Communities

**Figure 2:**
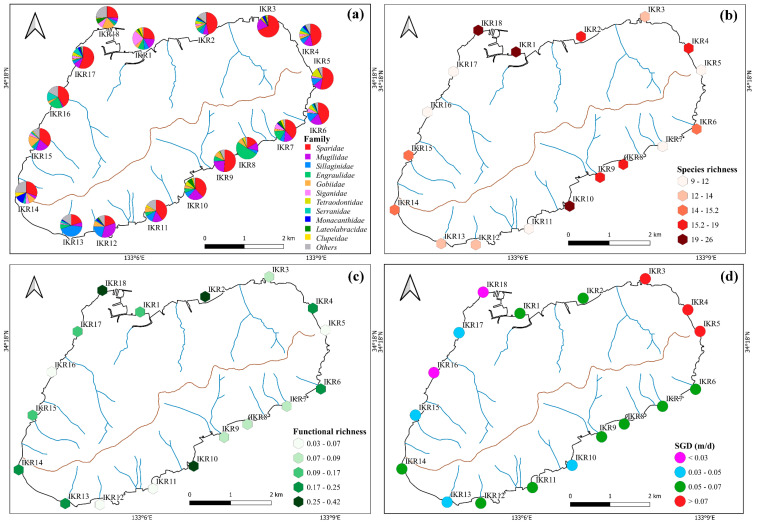
Spatial variation in the percentages of (**a**) fish family, (**b**) species richness, (**c**) functional richness and (**d**) SGD (point color relate to the SGD level of each stations: red for SGD value > 0.07 m/d, green for SGD value range 0.05–0.07 m/d, blue for SGD value range 0.07–0.05 m/d, and purple for SGD value < 0.03).

A total of 2,827,847 sequences clustered into 326 ZOTUs were detected by eDNA metabarcoding, which belong to 19 orders, 34 families and 59 species (Table S1). Most of the sequence reads were assigned to *Sparidae* (37%), followed by *Mugilidae* (14%), *Sillaginidae* (9%), *Engraulidae* (8%), *Gobiidae* (6%), and *Siganidae* (5%). Among them, *Sparidae*, *Mugilidae*, *Sillaginidae*, and *Gobiidae* were found frequently in the sampling stations; especially, *Sparidae* and *Mugilidae* had 100% occurrence frequency and were detected across all stations (Figure 2a). By contrast, *Haemulidae*, *Stichaeidae*, *Pristigasteridae*, *Scombridae, Sphyraenidae*, *Tripterygiidae*, *Hexagrammidae*, *Atherinidae*, and *Cynoglossidae* were found only once between different sampling stations (Figure S1). Our data showed that 30 fish species (belonging to 12 orders, 23 families) were identified from commercial to highly commercial in terms of fishery (Table S1). The alpha diversity index of fish diversity was found to be different across the sampling stations, suggesting a complex and heterogeneous distribution of fish communities within the island ecosystem (Figure 2b,c). Fish species richness and functional richness were highest in IKR1, 2, 4, 10 and 18, and the lowest in IKR11 and 16.

**Figure 3:**
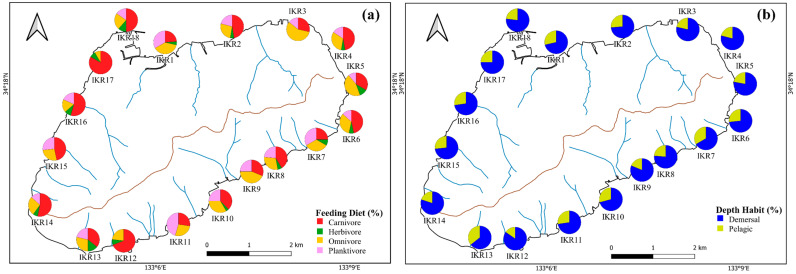
Spatial variation in the percentages of (**a**) feeding diet and (**b**) depth habit.

Based on feeding diet preference, fish were categorized into four groups, the major traits were carnivores (32 species), followed by omnivores (15 species), planktivores (7 species), and herbivores (3 species). Two depth traits were observed based on the trophic distribution of fish, which comprised 42 demersal, and 17 pelagic species. The distribution patterns of a functional fish community were dominated by fish that belonged to carnivores (54%) and with benthic habits (71% of demersal fish) (Figure 3).

### 3.2. Impacts of SGD on Fish Diversities

**Figure 4:**
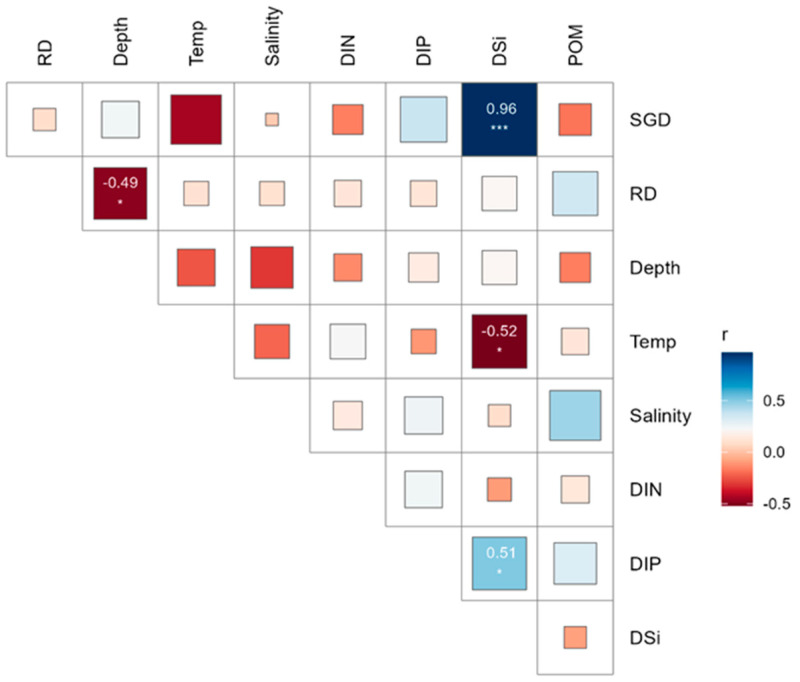
Spearman’s correlation matrix illustrates the relationships between environmental variables in the target island. Statistical significance markers: *** *p* < 0.001, * *p* < 0.05.

The temperatures recorded ranged from 19.8 °C to 21.7 °C, with a mean seawater temperature of 20.8 °C ± 0.5 SD (Standard Deviation). The average salinity was 33.5 psu ± 0.5 SD, ranging from 31.85 psu to 34.18 psu. Higher SGD occurred along the northeastern shoreline, and the lowest SGD was found along the northwestern shorelines (Figure 2d). The Spearman correlation analysis showed that SGD had a positive correlation with DSi and DIP concentrations and a negative correlation with water temperature and salinity, while RD had a positive correlation with POM (Figure 4). High positive correlations were observed among DSi and DIP, indicating that their sources were consistent and similar Figure 4).

The Alpha diversity index is an effective tool for characterizing the species diversity of Ikuchijima Island. In this study, higher species richness and functional richness were observed at stations with higher SGD, such as IKR2, 4, 6, and 14, indicating that SGD affects the composition of fish diversity within a small spatial island scale (Figure 2b,c).

Analysis of the effects of environmental variables on the pattern of fish diversity and communities showed that different species and functional traits of fish respond differently to environmental variables. The results of the best models in the GLMs are shown in Table 2. Carnivore richness had significantly negative relationships with salinity (*p* < 0.05), while planktivore richness and salinity had a positive relationship. RD and omnivore richness had a significant positive relationship (*p* < 0.05). These results suggest that the spatial patterns of fish diversity were influenced by different environmental variables.

**Figure 5:**
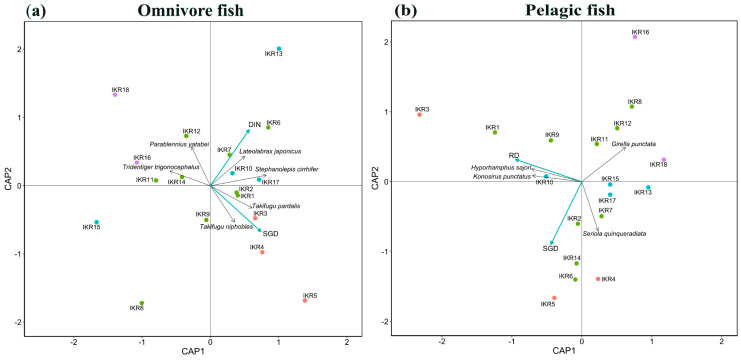
dbRDA results show model residual variables in (**a**) omnivore and (**b**) pelagic fish communities. Point colors relate to the SGD level of each station: red for SGD value > 0.07 m/d, green for SGD value range 0.05–0.07 m/d, blue for SGD value range 0.07–0.05 m/d, and purple for SGD value < 0.03. Blue arrows and black arrows represent environmental variables and fish species, respectively.

From the results of dbRDA, SGD and DIN concentration were key factors affecting the spatial composition of omnivore community (full model, F = 1.42, *p* < 0.05) (Figure 5a). SGD showed a strong significant effect on omnivore community composition (SGD, F = 1.46, *p* < 0.05). *Takifugu pardalis* and *Takifugu niphobles* were found to be highly occurring at high SGD sites. DIN concentration also had a strong significant effect on omnivore community (DIN, F = 1.38, *p* < 0.05). *Lateolabrax japonicus* and *Stephanolepis cirrhifer* were highly detected in high DIN sites. For pelagic community composition, RD and SGD were key variables affecting the community composition (full model, F = 1.34, *p* < 0.05) (Figure 5b). RD had a strong significant effect on pelagic community composition (RD, F = 1.56, *p* < 0.05). *Konosirus punctatus* and *Hyporhamphus sajori* were commonly observed in high RD sites.

## 4. Discussion

To our knowledge, this is the first study to address questions of spatial patterns of coastal fish communities along with variability of SGD and other environmental factors, using a combination of taxonomic and functional approaches at the coastal-island scale. In this study, we found that the functional group composition of coastal fish is greatly influenced by several variables, such as SGD, RD, salinity, and DIN concentration. Especially, the difference in salinity associated with SGD may affect the spatial distribution of fish through their choice of habitat. The positive correlation between SGD, DSi, and DIP concentrations also provides evidence of the SGD-derived nutrient supply to coastal seawater. Thus, SGD can provide physico-chemical beneficial environmental conditions for the growth and survival of fish communities. For example, the recorded conditions at station IKR2 are characterized by high SGD and RD, coupled with low salinity, as well as elevated levels of nutrients and POM.

Generally, the freshwater SGD can reduce salinity and may act as a buffer against elevated seawater salinity [6,9]. Different fish species prefer different ranges of salinity for growth, survival, feeding, breeding, nursing, etc., throughout their life cycle. We suggest that. at a small spatial scale, varying salinities influenced the spatial distribution of fish diversity within the target study. Additionally, Hata et al. [53] confirmed a high abundance of small crustaceans near the sites with high SGD, which is an important food resource for secondary consumers. Similarly, the high occurrence of omnivore-demersal species, such as panther puffer (*Takifugu pardalis*) and grass puffer (*Takifugu niphobles*), at the high SGD sites in this study suggested a similar causality in a previous study in northern Japan [54]. On the other hand, Kim et al. [55] reveal that RD had an impact on the contribution of the phytoplankton community, which may have influenced higher-trophic consumers, such as planktivore pelagic fish (*Konosirus punctatus*) and omnivore pelagic fish (*Hyporhamphus sajori*) in this study. Besides, the DIN/DIP and DIN/DSi ratios in the seawater of the target area are lower than the Redfield ratio, indicating that primary production is a nitrogen-limited condition [29]. Over recent decades, nutrient concentrations in the SIS have significantly shifted due to comprehensive efforts to reduce anthropogenic nutrient inputs, guided by the Total Pollutant Load Control System initiated in 1979 [56,57,58]. As a result, the SIS is currently characterized by oligotrophic conditions [59,60,61]. Several studies have identified nutrient scarcity resulting from oligotrophication as a critical factor in the declining fishery yields in the SIS [30,58,60]. Tanda and Harada [62] have revealed that reductions in nutrient levels are correlated with the fluctuation in the production of small pelagic and demersal fish species.

Besides, this study highlighted the advantages of the eDNA method, which provides a more comprehensive detection of fish species across an entire island than traditional methods, which have limitations in covering large areas [63,64,65]. According to Nhat et al. [29], eDNA technology can achieve similar or better results in assessing fish diversity than traditional monitoring methods. In this study, we demonstrated the feasibility of rapid marine biodiversity sampling using eDNA combined with functional traits to characterize the composition of coastal island ecosystems. Furthermore, eDNA can be used to capture the spatial distribution of fish communities within a coastal area. Several studies have shown that eDNA signals are localized in marine environments, leading to spatial patterns of fish diversity [20,21]. Here, we observed spatial variations in taxonomic and functional patterns of fish communities based on the localized signals of eDNA in the target area. Therefore, these results suggest that eDNA can provide a solution for rapid application in coastal monitoring of the spatial variability of fish diversity.

However, our study still had a few limitations. While eDNA is a valuable tool for obtaining species information, its current application is constrained in providing insights into the developmental stages of the detected organisms. Moreover, although multiple physico-chemical parameters were analyzed to understand the drivers of fish community composition, this investigation did not encompass the impact of associated communities, such as invertebrates and phytoplankton, which were not monitored during the study period. Future studies should consider developing a hybrid monitoring approach that integrates frequent eDNA surveys with traditional methods, such as fishing, Underwater Visual Census (UVC), and Baited Remote Underwater Video (BRUV). For example, combining eDNA metabarcoding with visual census methods and food web structure analysis (e.g., model analysis, stable isotope analysis) could offer a comprehensive overview of local fish composition and facilitate the exploration of relationships between fish assemblages and benthic communities. This integrated approach would enable more consistent and holistic monitoring of aquatic ecosystems. Incorporating historical survey data can enhance the precision of eDNA assays and provide a robust framework for interpreting the results. By addressing these limitations and adopting a more integrated monitoring approach, future research can achieve a more detailed and accurate understanding of the diversity and structure of fish communities, thereby improving the effectiveness of conservation and management strategies for coastal ecosystems.

## 5. Conclusions

In conclusion, this study demonstrated that the spatial patterns of diversity and composition of fish communities were related to the patterns of environmental variables. SGD is well recognized as one of the important factors that shape fish functional communities. This study also confirmed the usefulness of the eDNA method for assessing fish distribution and diversity in coastal ecosystems. By integrating taxonomic and functional diversity, eDNA monitoring provides complementary perspectives on community functions, thereby enhancing the overall assessment of community composition. To achieve a more detailed understanding of fish community diversity and structure, future research should incorporate eDNA methods alongside traditional monitoring techniques and consider a broader range of ecological variables. This integrated approach will offer a more comprehensive understanding of the factors shaping fish communities and improve the effectiveness of coastal ecosystem monitoring.

## Figures and Tables

**Table 1:** Trait groups and modalities were used in the study, with the indication of the category definition and the explanation for trait selection.

Table	Category	Definition	Rationale
Feeding diets	Carnivores	Feeding mainly on smaller fish	Defined based on fish diet and aggregation of fish species that utilize the same food resource [39]
	Herbivore	Feeding mainly on plant material	
	Omnivore	Feeding on food both of plant material and animal origins	
	Planktivore	Feeding mainly on plankton	
Depth habits	Pelagic	Inhabit the upper water column	Reflects the degree of dependence of fish on their position in the water column [40]
	Demersal	Live near the bottom	

**Table 2:** Summary of results of the best models of GLMs used to assess the relationship between the alpha diversity index and environmental variables.

Model	Variable (Intercept)	Salinity	SGD	RD	df	AIC
Carnivore richness	13.76 ***	−0.34 *	−7.5		3	85.4
Omnivore richness	1.62 ***			0.05 *	2	75.2
Planktivore richness	−20.55	0.64 *			0.05	76.2

AIC: Akaike’s information criterion. Statistical significance markers: *** *p* < 0.001, * *p* < 0.05 in a Wald test.

## Data Availability

The raw data supporting the conclusions of this article will be made available by the corresponding author on request.

## Supplementary Materials

The following supporting information can be downloaded at: https://www.mdpi.com/article/10.3390/biology13080609/s1, Figure S1: Heatmap of relative abundance (%) of fish family by eDNA metabarcoding at each station. The heatmap colors represent the relative abundances of fish species. Sorting is in proportion to relative abundance, with a decreasing trend from top to bottom; Table S1: Fish information in the target island.
