# Supplementary material for: Environmental DNA Reveals the Impact of Submarine Groundwater Discharge on the Spatial Variability of Coastal Fish Diversity
Source: Biology (Basel). 2024 Aug 11;13(8):609. doi: 10.3390/biology13080609 (PMC11351404; doi:10.3390/biology13080609)
Supplement: Supplementary file 1 [file biology-13-00609-s001.zip › biology-3131849-supplementary.pdf]

Supporting Information for "Environmental DNA reveals the impact of submarine groundwater discharge (SGD) on the spatial variability of coastal fish diversity"

Hong Nhat Nguyen[1,2], Mitsuyo Saito[3,*], Shin-ichi Onodera[3], Mayuko Hamada[4], Fujio Hyodo[5], Hideaki Nagare[5]

[1]*Graduate School of Environmental and Life Science, Okayama University, Okayama 7008530, Japan ;p1qd3i9i@s.okayama-u.ac.jp*

[2]*Faculty of Technology – Engineering – Environment, An Giang University, Vietnam National University Ho Chi Minh City, Long Xuyen 880000, An Giang, Vietnam*

[3]*Graduate School of Advanced Science and Engineering, Hiroshima University, Higashi Hiroshima 7398521, Japan ; sonodera@hiroshima-u.ac.jp*

[4]*Ushimado Marine Institute (UMI), Graduate School of Environment, Life, Natural Science and Technology, Okayama University, Okayama 7014303, Japan ; hamadam@okayama-u.ac.jp*

[5]*Faculty of Environmental, Life, Natural Science and Technology, Okayama University, Okayama 7008530, Japan ; fhyodo@cc.okayama-u.ac.jp (F.H.); nagare-h@okayama-u.ac.jp (H.N.)*

Corresponding author

*Email: misaito@hiroshima-u.ac.jp; Phone: +81-82-424-6521

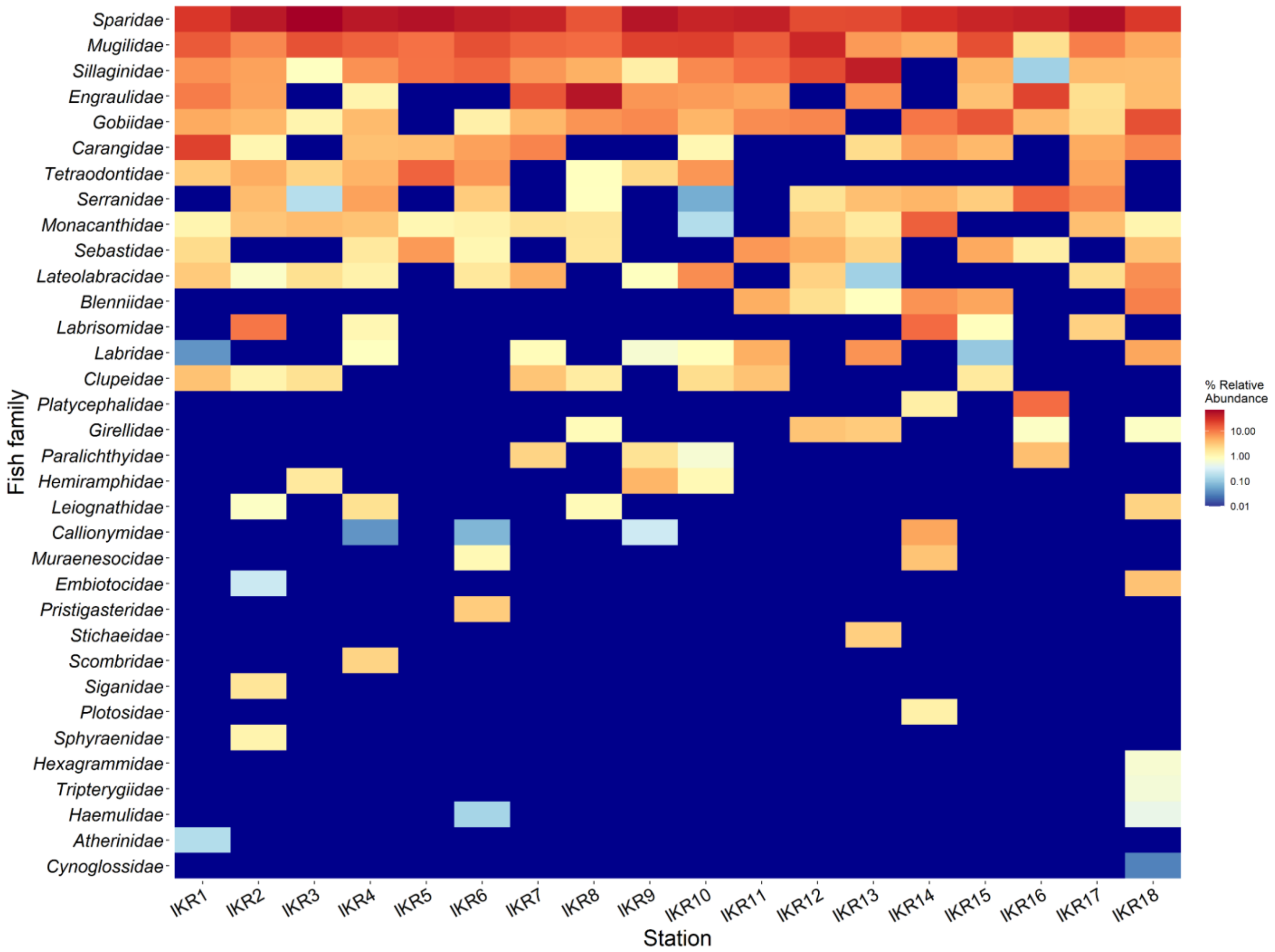

Figure S1. Heatmap of relative abundance (%) of fish family by eDNA metabarcoding at each station. The heatmap colors represent the relative abundances of fish species. Sorting is in proportion to relative abundance, with a decreasing trend from top to bottom.

Table S1. Fish information in the target island

ID	Class	Order	Family	Scientific Name	Depth Habit	Feeding Diet	Importance in Fisheries	IUCN Red List Status
1	*Actinopteri*	*Gobiiformes*	*Gobiidae*	*Acanthogobius flavimanus*	demersal	carnivore	commercial	Not Evaluated
2	*Actinopteri*	*Spariformes*	*Sparidae*	*Acanthopagrus latus*	demersal	carnivore		Data Deficient
3	*Actinopteri*	*Spariformes*	*Sparidae*	*Acanthopagrus schlegelii*	demersal	omnivore	commercial	Least Concern
4	*Actinopteri*	*Gobiiformes*	*Gobiidae*	*Acentrogobius pflaumii*	demersal	omnivore		Not Evaluated
5	*Actinopteri*	*Gobiiformes*	*Gobiidae*	*Apocryptodon punctatus*	demersal	omnivore		Least Concern
6	*Actinopteri*	*Atheriniformes*	*Atherinidae*	*Atherinomorus lacunosus*	pelagic	planktivore	commercial	Least Concern
7	*Actinopteri*	*Syngnathiformes*	*Callionymidae*	*Callionymus lunatus*	demersal	carnivore		Not Available
8	*Actinopteri*	*Syngnathiformes*	*Callionymidae*	*Callionymus valenciennei*	demersal	carnivore		Not Evaluated
9	*Actinopteri*	*Perciformes*	*Platycephalidae*	*Cociella crocodilus*	demersal	carnivore		Least Concern
10	*Actinopteri*	*Pleuronectiformes*	*Cynoglossidae*	*Cynoglossus interruptus*	demersal	carnivore	commercial	Not Evaluated
11	*Actinopteri*	*Carangiformes*	*Carangidae*	*Decapterus maruadsi*	pelagic	carnivore	highly commercial	Least Concern
12	*Actinopteri*	*Perciformes*	*Stichaeidae*	*Dictyosoma burgeri*	pelagic	carnivore		Not Evaluated
13	*Actinopteri*	*Perciformes*	*Embiotocidae*	*Ditrema temminckii*	demersal	carnivore	commercial	Not Evaluated
14	*Actinopteri*	*Clupeiformes*	*Engraulidae*	*Engraulis japonicus*	pelagic	planktivore	highly commercial	Least Concern
15	*Actinopteri*	*Perciformes*	*Serranidae*	*Epinephelus akaara*	demersal	carnivore	minor commercial	Endangered
16	*Actinopteri*	*Chaetodontiformes*	*Leiognathidae*	*Equulites rivulatus*	pelagic	carnivore		Not Evaluated
17	*Actinopteri*	*Gobiiformes*	*Gobiidae*	*Favonigobius gymnauchen*	demersal	carnivore		Not Evaluated
18	*Actinopteri*	*Centrarchiformes*	*Girellidae*	*Girella punctata*	pelagic	herbivore	commercial	Not Evaluated
19	*Actinopteri*	*Perciformes*	*Hexagrammidae*	*Hexagrammos stelleri*	demersal	carnivore	minor commercial	Not Evaluated
20	*Actinopteri*	*Beloniformes*	*Hemiramphidae*	*Hyporhamphus sajori*	pelagic	omnivore	commercial	Not Evaluated
21	*Actinopteri*	*Clupeiformes*	*Pristigasteridae*	*Ilisha elongata*	pelagic	planktivore	highly commercial	Least Concern
22	*Actinopteri*	*Gobiiformes*	*Gobiidae*	*Istigobius hoshinonis*	demersal	carnivore		Not Evaluated
23	*Actinopteri*	*Clupeiformes*	*Clupeidae*	*Konosirus punctatus*	pelagic	planktivore	minor commercial	Least Concern
31	*Actinopteri*	*Pempheriformes*	*Lateolabracidae*	*Lateolabrax japonicus*	demersal	omnivore	commercial	Not Evaluated
24	*Actinopteri*	*Mugiliformes*	*Mugilidae*	*Mugil cephalus*	pelagic	planktivore	highly commercial	Least Concern
25	*Actinopteri*	*Anguilliformes*	*Muraenesocidae*	*Muraenesox cinereus*	pelagic	carnivore	highly commercial	Not Evaluated
26	*Actinopteri*	*Gobiiformes*	*Gobiidae*	*Myersina filifer*	demersal	planktivore		Least Concern
27	*Actinopteri*	*Blenniiformes*	*Labrisomidae*	*Neoclinus chihiroe*	demersal	carnivore		Least Concern
28	*Actinopteri*	*Chaetodontiformes*	*Leiognathidae*	*Nuchequula nuchalis*	demersal	carnivore		Not Evaluated
29	*Actinopteri*	*Blenniiformes*	*Blenniidae*	*Omobranchus elegans*	demersal	omnivore		Least Concern

30	*Actinopteri*	*Blenniiformes*	*Blenniidae*	*Omobranchus elongatus*	demersal	omnivore		Least Concern
32	*Actinopteri*	*Blenniiformes*	*Blenniidae*	*Omobranchus punctatus*	demersal	omnivore		Least Concern
33	*Actinopteri*	*Spariformes*	*Sparidae*	*Pagrus major*	demersal	carnivore	highly commercial	Least Concern
34	*Actinopteri*	*Blenniiformes*	*Blenniidae*	*Parablennius yatabei*	demersal	omnivore		Least Concern
35	*Actinopteri*	*Gobiiformes*	*Gobiidae*	*Parachaeturichthys polynema*	demersal	carnivore		Least Concern
36	*Actinopteri*	*Labriformes*	*Labridae*	*Parajulis poecilepterus*	demersal	carnivore	commercial	Least Concern
37	*Actinopteri*	*Pleuronectiformes*	*Paralichthyidae*	*Paralichthys olivaceus*	demersal	carnivore	highly commercial	Not Evaluated
38	*Actinopteri*	*Mugiliformes*	*Mugilidae*	*Planiliza haematocheilus*	demersal	omnivore	commercial	Not Evaluated
39	*Actinopteri*	*Perciformes*	*Platycephalidae*	*Platycephalus indicus*	demersal	carnivore	commercial	Not Evaluated
40	*Actinopteri*	*Lutjaniformes*	*Haemulidae*	*Plectorhinchus cinctus*	demersal	carnivore	commercial	Not Evaluated
41	*Actinopteri*	*Siluriformes*	*Plotosidae*	*Plotosus japonicus*	demersal	carnivore		Not Evaluated
42	*Actinopteri*	*Syngnathiformes*	*Callionymidae*	*Repomucenus ornatipinnis*	demersal	carnivore		Not Evaluated
43	*Actinopteri*	*Tetraodontiformes*	*Monacanthidae*	*Rudarius ercodes*	demersal	carnivore		Near Threatened
44	*Actinopteri*	*Clupeiformes*	*Clupeidae*	*Sardinella lemuru*	pelagic	planktivore	highly commercial	Not Evaluated
45	*Actinopteri*	*Clupeiformes*	*Clupeidae*	*Sardinella zunasi*	pelagic	planktivore	highly commercial	Data Deficient
46	*Actinopteri*	*Scombriformes*	*Scombridae*	*Scomberomorus niphonius*	pelagic	carnivore	highly commercial	Near Threatened
47	*Actinopteri*	*Perciformes*	*Sebastidae*	*Sebastes sp.*	demersal	carnivore	commercial	Not Evaluated
48	*Actinopteri*	*Perciformes*	*Sebastidae*	*Sebastiscus marmoratus*	demersal	carnivore		Data Deficient
49	*Actinopteri*	*Labriformes*	*Labridae*	*Semicossyphus reticulatus*	demersal	carnivore		Least Concern
50	*Actinopteri*	*Carangiformes*	*Carangidae*	*Seriola quinqueradiata*	pelagic	planktivore	highly commercial	Least Concern
51	*Actinopteri*	*Perciformes*	*Siganidae*	*Siganus fuscescens*	demersal	herbivore	commercial	Least Concern
52	*Actinopteri*	*Perciformes*	*Sillaginidae*	*Sillago japonica*	demersal	carnivore	commercial	Least Concern
53	*Actinopteri*	*Perciformes*	*Sphyraenidae*	*Sphyraena pinguis*	pelagic	carnivore	highly commercial	Not Evaluated
54	*Actinopteri*	*Blenniiformes*	*Tripterygiidae*	*Springerichthys bapturus*	pelagic	herbivore		Least Concern
55	*Actinopteri*	*Tetraodontiformes*	*Monacanthidae*	*Stephanolepis cirrhifer*	demersal	omnivore	highly commercial	Least Concern
56	*Actinopteri*	*Tetraodontiformes*	*Tetraodontidae*	*Takifugu niphobles*	demersal	omnivore		Least Concern
57	*Actinopteri*	*Tetraodontiformes*	*Tetraodontidae*	*Takifugu pardalis*	demersal	omnivore		Least Concern
58	*Actinopteri*	*Tetraodontiformes*	*Monacanthidae*	*Thamnaconus modestus*	demersal	omnivore		Least Concern
59	*Actinopteri*	*Gobiiformes*	*Gobiidae*	*Tridentiger trigonocephalus*	demersal	omnivore		Not Evaluated
